# Active Dendrites Enhance Neuronal Dynamic Range

**DOI:** 10.1371/journal.pcbi.1000402

**Published:** 2009-06-12

**Authors:** Leonardo L. Gollo, Osame Kinouchi, Mauro Copelli

**Affiliations:** 1 Laboratório de Física Teórica e Computacional, Departamento de Física, Universidade Federal de Pernambuco, Recife, Brazil; 2 Instituto de Física Interdisciplinar y Sistemas Complejos, Palma de Mallorca, Spain; 3 Faculdade de Filosofia, Ciências e Letras de Ribeirão Preto, Universidade de São Paulo, Ribeirão Preto, Brazil; Université Paris Descartes, Centre National de la Recherche Scientifique, France

## Abstract

Since the first experimental evidences of active conductances in dendrites, most neurons have been shown to exhibit dendritic excitability through the expression of a variety of voltage-gated ion channels. However, despite experimental and theoretical efforts undertaken in the past decades, the role of this excitability for some kind of dendritic computation has remained elusive. Here we show that, owing to very general properties of excitable media, the average output of a model of an active dendritic tree is a highly non-linear function of its afferent rate, attaining extremely large dynamic ranges (above 50 dB). Moreover, the model yields double-sigmoid response functions as experimentally observed in retinal ganglion cells. We claim that enhancement of dynamic range is the primary functional role of active dendritic conductances. We predict that neurons with larger dendritic trees should have larger dynamic range and that blocking of active conductances should lead to a decrease in dynamic range.

## Author Summary

Most neurons present cellular tree-like extensions known as dendrites, which receive input signals from synapses with other cells. Some neurons have very large and impressive dendritic arbors. What is the function of such elaborate and costly structures? The functional role of dendrites is not obvious because, if dendrites were an electrical passive medium, then signals from their periphery could not influence the neuron output activity. Dendrites, however, are not passive, but rather active media that amplify and support pulses (dendritic spikes). These voltage pulses do not simply add but can also annihilate each other when they collide. To understand the net effect of the complex interactions among dendritic spikes under massive synaptic input, here we examine a computational model of excitable dendritic trees. We show that, in contrast to passive trees, they have a very large dynamic range, which implies a greater capacity of the neuron to distinguish among the widely different intensities of input which it receives. Our results provide an explanation to the concentration invariance property observed in olfactory processing, due to the very similar response to different inputs. In addition, our modeling approach also suggests a microscopic neural basis for the century old psychophysical laws.

## Introduction

One of the distinctive features of many neurons is the presence of extensive dendritic trees. Much experimental and computational work has been devoted to the description of morphologic and dynamic aspects of these neural processes [1], in special after the discovery of dendritic active conductances [2]–[4]. Several proposals have been made about possible computational functions associated to active dendrites, such as the implementation of biological logic gates and coincidence detectors [5],[6], learning signaling via dendritic spikes [7] or an increase in the learning capacity of the neuron [8]. However, it is not clear whether such mechanisms are robust in face of the noisy and spatially distributed character of incoming synaptic input, as well as the large variability in morphology and dendritic sizes.

Here we propose to view the dendritic tree not as a computational device, an exquisitely designed “neural microchip” [6] whose function could be dependent on an improbable fine tuning of biological parameters (such as delay constants, arborization size, etc), but rather as a spatially extended excitable system [9] whose robust collective properties may have been progressively exapted to perform other biological functions. Our intention is to provide a simpler hypothesis about the functional role of active dendrites, which could be experimentally tested against other proposals.

We study a model where the excitable dynamics is simple, but the dendritic topology is faithfully reproduced by means of a binary tree with a large number of excitable branchlets. Most importantly, branchlets are activated stochastically (at some rate), so that the effects of the nonlinear interactions among dendritic spikes can be assessed. We study how the geometry of such a spatially extended excitable system boosts its ability to perform non-linear signal processing on incoming stimuli. We show that excitable trees naturally exhibit large dynamic ranges — above 50 dB. In other words, the neuron could handle five orders of magnitude of stimulus intensity, even in the absence of adaptive mechanisms. This performance is one hundred times better than what was previously observed in other network topologies [10],[11].

Such a high performance seems to be characteristic of branched (tree) structures. We believe that these findings provide important clues about the possible functional roles of active dendrites, thus providing a theoretical background [4] on the cooperative behavior of interacting branchlets. We observe in the model the occurrence of dendritic spikes similar to those already observed experimentally and recently related to synaptic plasticity [7]. Here, however, such spikes are just an inevitable consequence of the excitable dynamics and we propose that even dendritic trees without important plasticity phenomena (like those of some sensory neurons) could benefit from active dendrites from the point of view of enlargement of its operational range.

Our results also suggest that, under continuous synaptic bombardment, dendritic spikes could be responsible for another unintended prediction of the model, namely, that the neuron transfer function needs not to be simply a Hill-like saturating curve; rather, a double-sigmoid behavior may appear (as observed experimentally in retinal ganglion cells [12]). The model further predicts that:

- the neuron average activity depends mainly on the rate of branchlet activation, reflecting in a robust way the afferent input, and not on the total number of branchlets present in the tree, which is highly dependent on accidental morphological details;
- the size of the dendritic arbor, or rather the number of bifurcations of the tree, affects in a specific manner the neuronal dynamic range.

So, why do neurons have active dendrites? As a short answer, we propose that neurons are the only body cells with large dendrites because they need to work with a large stimulus range. Owing to the enormous number of afferent synapses and the large variability of input rates, highly arborized and *active* dendrites are crucial to enhance the dynamic range of a neuron, in a way not accounted for by passive cable theory and biophysical neuron models with few compartments (reduced models) [13]. Other phenomena, such as backpropagating spikes, could have been later exapted to more complex functional roles. One should, however, consider first a generic property of extended excitable media: that, due to the creation and annihilation of non-linear pulses, the input-output transfer function of such media is necessarily highly non-linear, with a very large dynamic range as compared with that of a passive medium.

## Methods

### Modeling active dendrites

In computational neuroscience, the behavior of an active neuronal membrane traditionally is modeled by coupled differential equations which represent the dynamics of its electric potential and gating variables related to the ionic conductances. This modeling strategy was then further extended by detailing the dendritic tuft through a compartmental approach [14]. Motivated by the abundant evidence that dendrites have active ion channels that can support non-linear summation and dendritic spikes [1],[6], this line of research currently aims at examining the possibility that these extensive tree-shaped neuronal regions may be the stage for some kind of “dendritic computation” [4],[5],[15].

Many efforts within this framework of biophysical modeling have been devoted to unveiling the conditions under which the regenerative properties of dendritic active channels may be unleashed to generate a nonlinear excitation (e.g. at the level of a single spine [16] or upon temporally synchronized and locally strong input at the level of a branchlet [17]). Nonlinear cable theory can further help predict whether and how a single dendritic spike will propagate along the branches, for instance highlighting the relative importance of a given channel type for the propagation of action potentials [18]. Detailed biophysical models also correctly predicts e.g. that two counter-propagating dendritic spikes annihilate each other upon collision [19],[20] (instead of summing), but this is true for most – if not all – extended excitable media. However, at the present state of the art of neuronal simulations, biophysical modeling may not necessarily be the approach best suited for addressing the much more difficult question of what happens when many dendritic spikes interact, specially in a more natural scenario where they would be continuously created at different points of the dendritic tree at some stochastic rate.

Understanding the net effect of the creation and annihilation of dendritic nonlinear excitations under massive spatio-temporal patterns of synaptic input requires 1) knowledge of the key properties of these excitations and their interactions (which cable theory gives us) and 2) a theoretical framework which addresses the resulting collective behavior. We therefore borrow from cable theory the facts that dendritic spikes may (or may not) be created by integrated synaptic input at some branchlets, then may (or may not) propagate to neighboring branchlets, and annihilate upon collision owing to refractoriness. Then, by employing a simplified excitable model for each branchlet, but a realistic multicompartment dendritic tree, we are able to focus on their collective behavior and to cast the dynamics of the dendritic tuft into the framework of extended excitable media, where both numerical and theoretical approaches have been successfully applied [9]–[11], [21]–[27].

Conventional wisdom in computational neuroscience is that in the limit of a very large number of compartments the model would be physically accurate. But in this same limit, conventional wisdom in statistical physics (say, renormalization group arguments) tells us that collective behaviors should be very weakly dependent on the detailed modeling of the basic (compartmental) unit [28]. Macroscopic properties of extended media would rather depend more strongly on dimensionality, network topology, symmetries, presence of parameter randomness (disorder), noise, boundary conditions etc. Therefore modeling should concentrate efforts on these more decisive aspects, the use of simple excitable dynamics for the elementary units being justified as a first approximation.

#### Elementary dynamics

**Figure 1:**
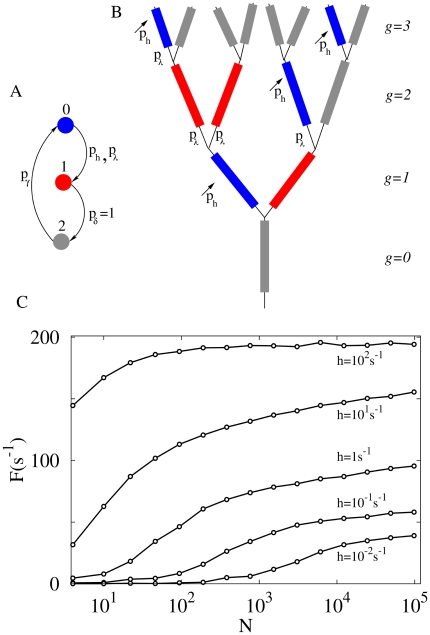
Morphology and dynamics of the model. (A) Definition of dynamical states: each dendritic branchlet can be in one of three states (represented by circles): quiescent (blue), active (red) or refractory (grey). A quiescent state becomes active due to integrated synaptic input (with probability 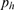) or transmission from an active neighbor (with probability 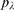, also called the coupling parameter). The active state has a fixed duration, changing to the refractory state after a single time step (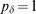). The refractory state returns to the quiescent state with probability 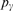 ( = 0.5 unless otherwise stated). (B) Example of an active dendritic tree with 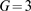: branchlets connected in a binary tree topology. The probability that activity in one branchlet activates its neighbour is 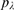 (if the neighbor is in a quiescent state). (C) Apical activity 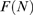 as a function of the number 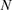 of dendritic branchlets. Due to integrated synaptic input, each branchlet becomes excited with a probability distribution modeled as an independent Poisson process with rate *h*, as well as deterministic propagation from active neighbors (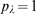). From bottom to top: 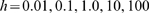 activations per second at each branchlet.

To account for the active nature of dendritic branchlets, each site is modelled as a simple discrete excitable element: 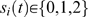 denotes the state of site 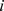 at time 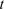 (Fig. 1A). If the 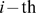 branchlet is active (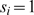), in the next time step it becomes refractory (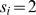). The average refractory period is controlled by 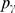, which is the probability with which sites return to a quiescent state (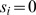) again. Only quiescent sites can become active due to activation by neighboring compartments or by external (synaptic) inputs. Owing to the sometimes small density of ionic channels in dendrites, transmission of excitations from active to quiescent sites is modelled to occur with probability 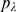 per bond (see Fig. 1B). This means that, in the model, the propagation of excitation from branchlet to branchlet is not deterministic and may fail with probability 1−*p_λ_*.

#### Dendritic topology

We can think of an active dendritic tree as an excitable network in which each site represents, for instance, an excitable dendritic branchlet connected with two more distal sites and one more proximal site (see Fig. 1B). That is, each branch at “generation” 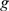 has a mother branch from “generation” 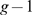 and gives rise to two daughter branches at “generation” 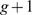 (i.e. each site — except those at the borders — has three neighbors). The most distal generation will be called level 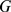 and we will study tree properties as a function of the branching order 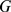. The single site at 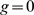 would correspond to the primary dendrite which connects with the neuron soma (see Fig. 1B). Notice that the number 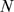 of branchlets grows exponentially with the branching order *G*.

#### External stimuli and branchlet activation

Each branchlet receives a large number of synapses, whose post-synaptic potentials (excitatory and inhibitory) are integrated. The final outcome of this complex integration (which we do not model here) may or may not trigger a branchlet spike, which we denote as our 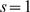 active state. As a first approximation, we assume that this branchlet activation process (or crossing of the excitability threshold) is Poisson with rate *h*, which somehow reflects the average excess of excitation as compared to inhibition. Thus, besides transmission from active neighbors, each quiescent branchlet can independently become active with probability 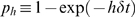 per time step (see Fig. 1B), where 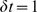 ms is an arbitrary time step.

We assume that the activation processes of different branchlets are independent of one another. Besides, we consider first the uniform case where all branchlets have the same excitation rate 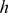, which is perhaps a reasonable assumption e.g. for mitral cells in the olfactory system [29]. We recognize that these are strong simplifications, but the analysis of this case is essential as a first step. Later we discuss non-homogeneous cases where 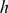 depends on the generation 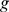 or, for each branchlet, is drawn from a normal distribution.

### Dendritic tree output as a response function

We define the apical activity 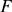 as the number of excitations (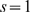 states) produced at the 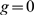 site, averaged over a large time window (10^4^ time steps and five realizations, unless otherwise stated). In the following we will be interested in understanding the function 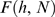, which is somehow analogous to the neuron frequency versus injected current 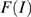 curves studied in the neuroscience literature. We suppose that, in the absence of lateral inhibition, the neuron firing frequency produced at the axonal trigger zone will be proportional to the apical activity 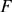, which is assumed by some biophysical models [30] and supported by recent experimental evidence in the *Drosophila* olfactory system [31].

For readers familiar with statistical physics models we observe that 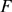 is the order parameter and 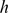 is an external field that drives the system to an active state with 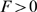. Our model is an out-of-equilibrium system with one absorbing state [32]. This means that, in the absence of external drive (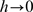) the dynamics eventually takes the system to a global resting (quiescent) state 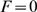 from which it cannot escape without further external stimulation. In biological terms, this simply means that our dendritic tree will not show spontaneous dendritic spikes without external synaptic input and any activity in the tree will eventually die if 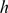 is turned to zero.

## Results

### Output dependence on arbor size 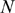


Dendritic trees are responsible for processing incoming stimuli which impinge continuously on the many synaptic buttons spread on the dendrites (a single olfactory mitral cell can have around 30,000 synapses, whereas cerebellar Purkinje cells have around 200,000 synapses). Of course these numbers vary also between individual cells of the same type. So, we first consider a classical question asked (and not clearly answered) in the literature: given a constant activation 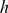 in cells with different arbor sizes, will they fire at very different levels [33]? The answer is not obvious since they may have a huge difference of absolute number of synapses and branchlets and we could have the prejudice that cells that have more synapses should fire more easily (or at least need to implement some homeostatic mechanism for controlling their firing rate).

The answer provided by our model is very interesting: for low excitation rate *h*, the output 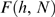 increases linearly with the number 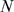 of branchlets, so that having a large branched tree is indeed important to amplify very weak signals (see Fig. 1C). In this context there is a clear reason for a neuron to maintain a costly number of branchlets. However, for moderate and high activation levels, the activity 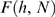 depends very weakly on 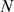 (it grows sub-logarithmically with *N*, see Fig. 1C for 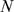, say, larger than 5,000). That is, in this regime the output 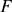 reflects, in an almost size-independent way, mostly the Poisson rate *h*, not the absolute number of branchlets activated on the tree.

Large dendritic arbors therefore aid the detection of weak stimuli, but for higher activation levels (i.e. higher imbalance between excitatory and inhibitory signals) all the neurons code in a similar way the activation rate 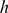, irrespective of their arbor size. Note that this “size invariance” is an intrinsic property of the excitable tree, not based on any homeostatic regulatory mechanism [19],[34]. This sublogarithmic dependence of 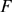 on 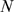 means that neurons function as reliable transductors for the signal 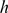: the specific number of branchlets, developmental defects, or asymmetries of the dendritic tuft have only a secondary effect in the global neuron functioning.

### Output dependence on excitation rate 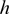


Given that the cell output depends weakly on 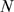, now we turn our attention to how 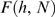 depends on the activation rate 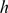. Note that not much modeling work has been done on addressing the collective activity of the dendritic tree subjected to extensive and distributed synaptic input [35],[36], particularly as the activation rate 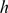 is varied. However, this is one of the simplest questions one may ask regarding dendritic signal processing. In particular, studies with models where the whole dendritic tree is reduced to a small number of compartments (reduced compartmental models [37]) can hardly address this issue, since the complex spatio-temporal information of the tree activation is lost by definition.

**Figure 2:**
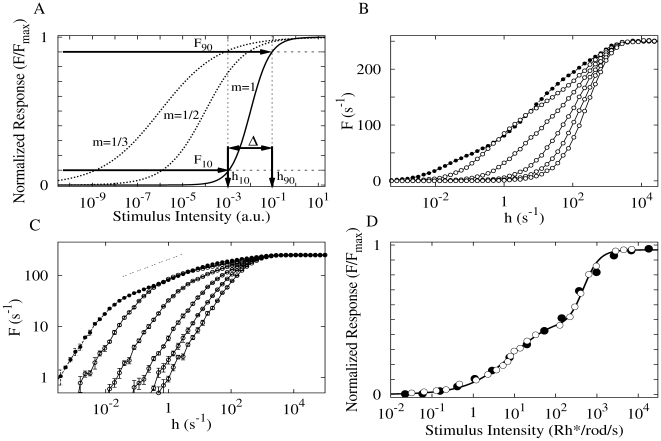
Response functions 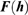. (A) Response functions exemplified by normalized Hill functions 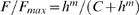 with different Hill exponents *m*. Relevant parameters for calculating the dynamic range are exemplified for 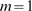, in which case 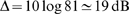 (see Eq. 1 and text for details). (B) Family of response curves 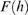 for 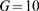. From bottom to top, open symbols represent 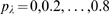, whereas closed symbols represent a deterministic transmission of activity (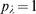) between dendritic branchlets. For 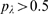 extra inflection points appear, giving rise to double sigmoid functions. (C) Study of the response exponent. Same curves as (B), but in double logarithmic scale. Notice the emergence of a non trivial and very small exponent 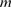 (≈0.2, thin dashed line) when reliability of dendritic spike propagation (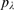) increases. Notice also that, for small input, spikes seldom colide: the output frequency 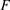 is thus proportional (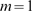) to the rate of branchlet activation (which creates the spikes). (D) Double sigmoid experimental response curve of retinal ganglion cells extracted from Ref. [12] (open symbols) compared to simulation results (closed symbols) for 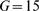 and 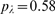. To scale the model variable 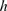 (ms^−1^) to the experimental stimulus intensity 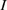 (Rh*/rod/s), we have employed 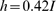. The solid curve is a fit of two different Hill functions joined together at 110 Rh*/rod/s.

As is well known, the average firing rate 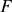 dependence on stimulus rate 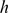 of several cells has a saturating aspect like that of Fig. 2A. Our cell presented a similar behavior (Fig. 2B), although of course it is not the simple Hill function 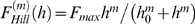 usually employed to fit experimental data. Indeed, for some values of axial transmission *p_λ_*, we saw an unexpected double-sigmoid behavior (see below).

#### Tree dynamic range

The dynamic range 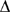 of the response function follows a standard definition:
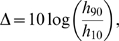
(1)where 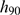 (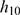) is the stimulus value for which the response reaches 90% (10%) of its maximum range (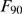 and 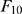 respectively). As exemplified in Fig. 2A with a Hill function, 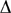 amounts to the range of stimulus intensities (measured in dB) which can be appropriately coded by 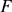, discarding stimuli which are either so weak as to be hidden by noise or self-sustained activity of the system (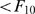) or so strong that response is in practice non-invertible owing to saturation (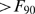). It is a straightforward exercise to show that for a general Hill function with exponent 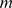 we have 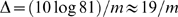 dB. This simple analytical result reinforces the fact that the exponent 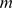 governing the low-stimulus regime is determinant for the dynamic range.

**Figure 3:**
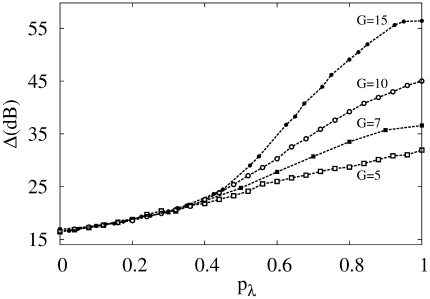
Enhacing the dynamic range. Dendritic trees perform non-linear input-output transformations such that the capacity to distinguish between different stimulus intensities, measured by the dynamic range (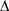) increases monotonically with coupling 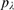 and the tree size (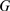). The tree topology can produce very large dynamic ranges (above 50 dB).

Figure 2B shows how the response curve changes with the coupling 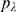 between dendritic patches. For 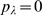 (lowest curve) each dendritic patch is an isolated excitable element, so activity does not spread in the tree and the response function is a linear-saturating curve with a small dynamic range (≃16 dB, see Fig. 3). As 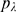 increases, more signals are transmitted to the apical site. This amounts to an amplifying mechanism whose efficiency increases with 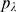, as depicted in Fig. 2B.

Amplification, however, is highly nonlinear. Note that a dendritic spike dies after some time either by propagation failure or, more importantly, because it is annihilated upon collision with other excitable pulses or with the tree boundaries (the 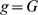 distal branches). Since the likelihood of these collisions increases with the stimulus intensity, amplification is stronger for weak stimuli and weaker for strong stimuli (in particular, for very strong stimulus every excitable element reaches its maximum activity – limited by refractoriness – and coupling is almost irrelevant). As a consequence, sensitivity *and* dynamic range are concurrently enhanced with increasing coupling, as illustrated in Fig. 3.

We emphasize that the above reasoning relies on very general and robust properties of excitable media: any detailed compartmental biophysical model of an active dendritic arbor will present similar results. Two features, however, strike as particularities of a tree topology: 1) the dynamic range attains extremely large values (see Fig. 3) and 2) the response functions can become double-sigmoids, due to interaction with dendritic backspikes, as depicted in the upper curves of Fig. 2B and discussed below.

## Discussion

A possible critique to our modeling approach is that it lacks biological realism. We notice that this is only true at the level of the biophysical dynamics of each compartment, but we believe that the idealization of such compartment as a generic excitable element (a cyclic automaton) is immaterial. This has already been demonstrated in studies of the dynamic range of networks composed by cellular automata, non-linear discrete time maps, nonlinear differential equations and conductance-based models (Hodgkin-Huxley compartments) [22],[27], as well as in a biophysically detailed model of the vertebrate retina [38].

Our model has realistic biological aspects not reproduced by most works in computational neuroscience with detailed biophysics:

- a tree topology with a very large number (≈10^5^) of compartments (branchlets);
- a proportionally large number of synaptic inputs;
- distributed activation along the whole tree instead of artificial injected currents applied at particular points.

Notwithstanding the fact that artificial input protocols like punctual current injection are useful for comparison with experimental measurements [39], we believe that spatio-temporal Poisson activation is a step toward a more realistic modeling of the dendritic arbor dynamics under natural circumstances [19],[35],[36].

### The dynamic range problem

As can be viewed in Fig. 3, large active dendritic trees perform strong signal compression, which is the ability of coding many orders of magnitude of stimulus intensity through only one decade of output frequency. This question is particularly important in sensory processing, where *many* orders of magnitude of stimulus intensity are present. Interestingly, olfactory glomeruli, constituted primarily by large active dendrites of mitral cells in vertebrates and dendrites of principal cells in insects have large dynamic range [40]–[42]. We conjecture that a similar situation occurs in the problem of fine motor control and sensory-motor integration in the cerebellum [43], which also involves the necessity of handling sensory-motor feedback signals varying by orders of magnitude. In correspondence to our hypothesis, Purkinje cells, which are involved in these tasks, have indeed enormous active dendritic arbors [13],[44].

Previous work [10], [11], [21]–[27] has shown that the non-linear summation of spikes enhance the dynamic range of excitable media. The tree topology, however, has not been studied in these works. Surprisingly, we found that its performance is largely superior to the others. This motivates the proposal, first made here (to the best of our knowledge), that the main functional role of active dendrites is to enlarge the cell dynamic range.

As a particular application, we discuss now the case of the dynamic range of olfactory glomeruli. Recent results for second-order projection neurons of the *Drosophila melanogaster* antennal lobe clearly exhibit strong weak-stimulus amplification and enhanced dynamic range as compared to olfactory receptor neurons (ORNs) [42]. To account for this observation, we can interpret our model as representing a *Drosophila* principal cell (analogous to a mitral cell) inside the glomerulus. Also, the signal propagation from ORN axons to principal cell dendrites and the proportionality between apical activity and somatic firing measured by Root *et al.* in the *Drosophila* is compatible with our identification of 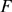 with the somatic neuron response [31] in this particular case. These authors show that it is mainly the ORN activity that drives the projection neuron firing rate, the isolated effect of synapses from interneurons (excitatory and inhibitory) being not sufficient to induce spikes and having mostly a modulatory role.

Of course, in the case of other biological systems like the mammalian olfactory bulb (where strong lateral inhibition occurs) or pyramidal cells, the identification of 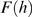 with the somatic firing rate is problematic, but we claim that the model is still useful for understanding of the large dynamic range (as measured by Calcium fluorescence) observed in the neuronal tuft [40],[41].

It is important to notice that large dynamic ranges as observed here means that the output varies slowly with the input. Therefore, if experiments are done over only one or two orders of magnitude of stimulus intensity (10–20 dB), the observed effect could be confounded with an almost constant response. This may be an alternative explanation for the *concentration invariance* property observed in olfactory processing [45].

### Weak dependence of activity on branchlet number

Another important prediction of our model is that dendritic size (and the respective number of branchlets and synapses) has a weak effect on the apical activation, being important mostly in the small excitation regime. It is mainly the branchlet activation rate *h*, not the total number of branchlets, that controls the apical rate *F*. This is a desirable robustness property since there is a high variability of dendritic size and spine density within a neuron population and along time in the same neuron.

Whichever function one wishes to assign to active dendrites, it must be fault tolerant in relation to gross dendrite morphology, branchlet excitability and synaptic density, which vary with age and time: for example, 30% of spine surface retracts in hippocampal neurons over the rat estrous cycle [46]. Due to the sublogarithmic dependence of 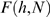 on 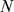 (see also the Model Robustness section), our model demonstrates that such gross independence from branchlet number, detailed branchlet dynamics, dendritic axial conductance and tree morphology is possible, and that enhancement of dynamic range is one of the most visible properties of these excitable trees.

### Response functions with double sigmoids

Double-sigmoid response functions have been reported recently for retinal ganglion cells of the mouse [12]. This unusual shape contrasts with the standard Hill fitting function. One wonders whether the habit of fitting Hill functions to data could have prevented further double-sigmoid curves from having been reported in the literature.

It is very interesting that such double sigmoid behavior is a distinctive feature of our model in a certain range of parameter space. Can we interpret the findings on retinal ganglionar cells in terms of our simplified model of dendritic response? Ganglionar cells have dendritic arbors but their size is small compared to, say, mitral cells or our typical model with branching order around 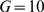.

However, in a structural analogy between the visual and olfactory systems, Shepherd proposed that some ganglionar cells are the retinal equivalent of mitral cells [44]. Here we pursue this analogy and suggest that the ganglionar dendritic arbor plus the retinal cells connected to it by gap junctions (electrical synapses) can be viewed as an extended active tree similar to the one studied here, with a large effective 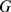.

We show in Fig. 2D that an appropriate choice of the model parameters can lead to a response function which fits the experimental data. Of course, the quantitative fit, although good, is not the important message, but the qualitative one: that double-sigmoid response functions can appear solely due to the tree topology, without invoking any secondary activation processes or complicated mechanisms to produce the unusual shape.

What is the physical origin of the double sigmoids in our model? We believe that it is related to the two different modes of activation of the apical site. The first one is the direct excitation due to its local 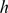 rate. This direct excitation, if large, drives the system to its maximum firing rate, which scales with the inverse of its refractory period. This mechanism would be responsible for the saturation in the right side of 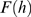 (region of large 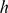), see Fig. 2B.

But the apical site also receives signals from its extended dendritic tree, which is very sensitive to small activity (extending the 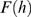 curve to the small 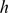 regime). However, it is plausible that the tree excitability saturates for a smaller frequency, due to the complicated interations between the spikes in the tree. So, we conjecture that the first sigmoid represents a bottleneck effect related to saturation in the flux of the activity along the subtrees connected to the apical site. Indeed, this is compatible with the observation that if we disconnect the apical site from the dendritic tree (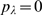, Fig. 2B), the double sigmoidal behavior disappears and only the second (large 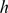) sigmoid is maintained. Of course, a more detailed analysis of the origin of the first sigmoid is needed.

We also observed curves with three sigmoids (see Model Robustness section), but postpone the discussion of these results to future works. We only note here that the 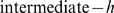 plateau in these curves could also be related to the concentration invariance reported for olfactory systems [45].

### Screening resonance

As can be seen in Fig. 2B, some response curves in our model can present an unusual shape, with curves for higher probability of axial transmission 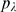 falling below curves for lower 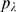. How can more efficient trees present a response below less efficient ones for the same 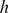 level?

**Figure 4:**
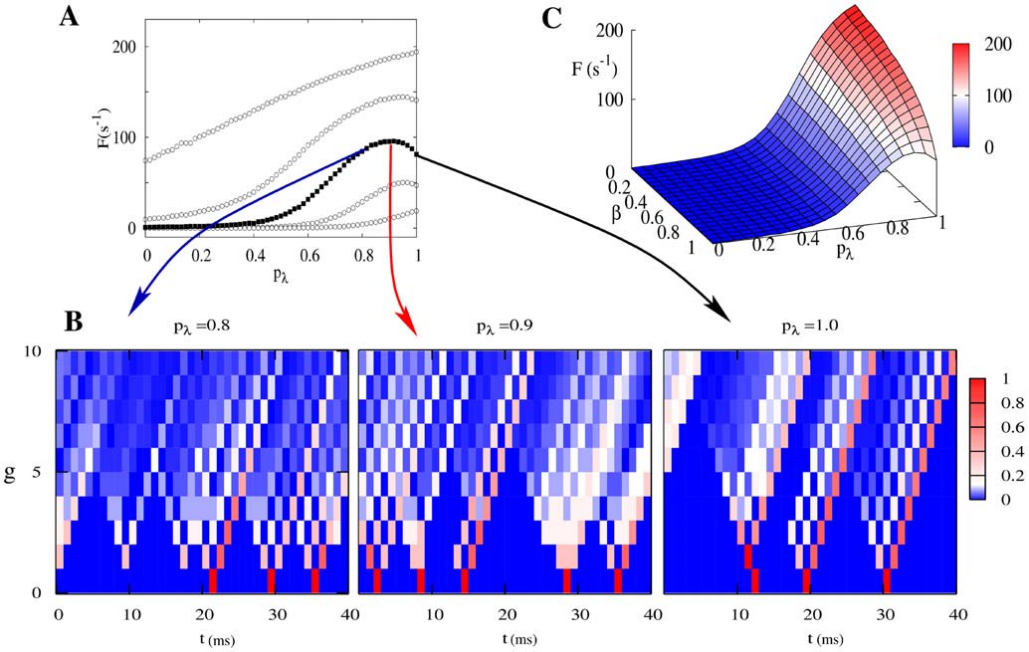
Screening resonance. Depending on the rate *h*, a maximum on the neuronal apical activity may be observed at an intermediate coupling value (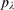). Propagation of forward signals fails to effectively induce neuronal apical activity for higher values of coupling due to backpropagating activity in a certain range 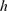 (here, the retropropagation ratio is 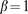). (A) The non-monotonous behavior in the mean output frequency 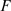 at the primary dendrite as a function of the coupling 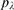 among sites (closed symbols). From bottom to top, 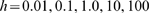 activations per second per branchlet. (B) Density of active branchlets at generation 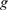 vs. time for 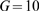 and 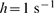. Notice that in this short (40 ms) sample, apical activity was higher for 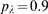 (5 activations) than for 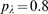 or 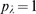 (3 activations each). The backpropagating signal for 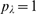 prevents distal activity from reaching the apical branchlet. (C) 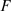 as a function of backpropagation ratio 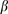 and coupling probability 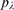, for fixed 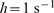: the screening resonance disappears in the absence of backspikes (low values of 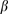).

This question can be answered by looking at Fig. 4A, where we plot a family of curves 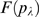 for fixed 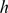. For some (intermediate) values of 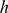, this curve is non-monotonic, suggesting a kind of resonance through which activity in the primary dendrite is maximized for an optimal coupling among sites all over the tree. Why is this so?

**Figure 5:**
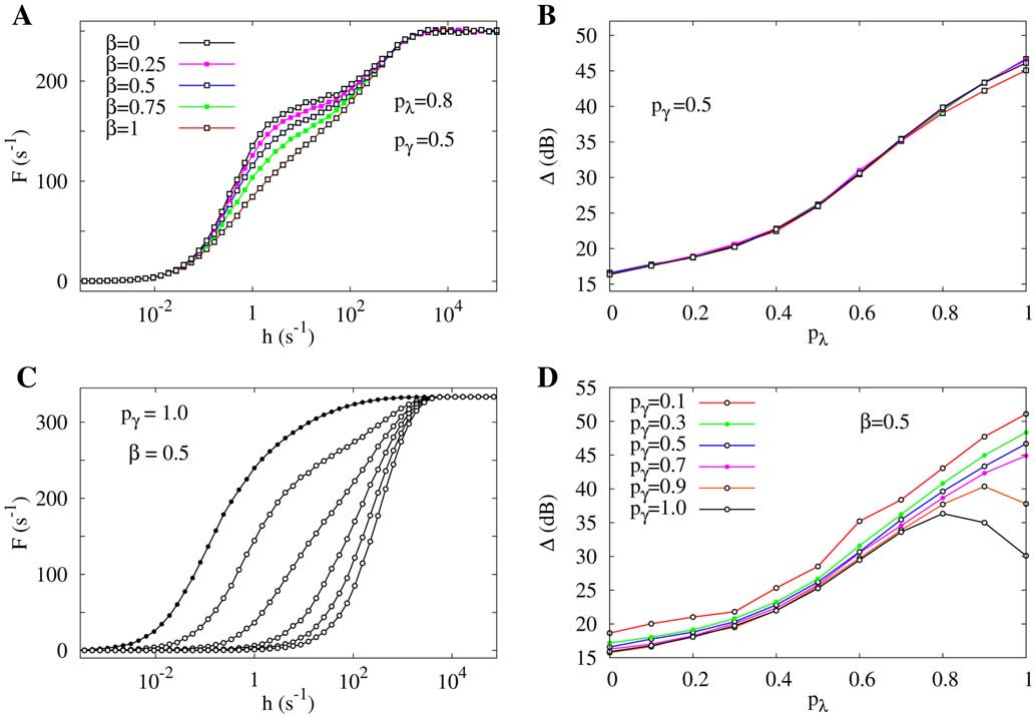
Effect of asymmetric propagation. (A) Response functions for different values of backpropagation ratio 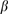 for fixed values of transmission probability 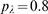 and recovery probability 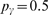 (which controls the refractory period). It shows a specific shape dependence with more visible double sigmoid behavior for less backspike activity (lower values of 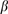). (B) Dynamic range of the response functions shown in panel (A). Although the response functions 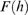 have different shapes, their dynamic ranges remain pretty much unaltered since the region in which the response functions differ is located in between the range of 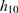 and 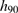 (see definition of dynamic range in Fig. 2A). (C) A family of response functions for deterministic refractory period (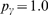) and asymmetric propagation (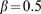). Similarly to Fig. 2B, from bottom to top open symbols represent 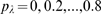, and filled circles represent the case of 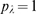. The model presents a wide variety of response function shapes. The filled symbols present a dynamic range smaller than for 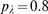, which is a rare example of non-monotonicity of the 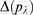 dependence. It occurs because the gain in sensitivity (of 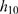) when 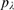 increases from 0.8 to 1 is less than what is lost due to an early saturation (of 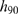). (D) Dynamic range for different values of refractory period. The black curve displays the non-monotonicity explained in panel C). Besides this feature (which occurs only for 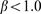) the dynamic range does not present qualitative changes compared to the standard symmetric model of Fig. 3.

Note that, on the one hand, for low enough 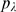, excitations created in distal sites may not arrive at the primary site due to propagation failure. For too strong coupling, on the other hand, *the topology* of the tree leads to a dynamic screening of the primary dendrite: backward propagation of activity (backspikes) effectively can block forward propagation of incoming signals, as shown in Fig. 4B. Activity 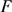 is therefore maximized at some intermediate value of coupling. We called this phenomenon “screening resonance”. That such screening resonance indeed depends on backspikes is confirmed by an asymmetrical propagation variant of the model (see Model Robustness section). As backpropagation goes to zero, the crossing between 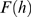 curves disappears (Figs. 4C and 5C).

The transmission probability 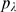 accounts for the joint effects of membrane axial conductance and density of regenerative ionic channels (Na^+^, Ca^2+^, NMDA etc). A possible experiment to test whether this screening resonance indeed exits could involve the manipulation of the density (or efficiency) of those channels in the dendritic tuft: the model predicts that more excitable trees may present lower activity than less excitable ones due to resonant annihilation of dendritic spikes.

### Testing dendritic spike annihilation

As discussed above, annihilation due to collision of dendritic spikes is the central mechanism in our model behind both the dynamic range enhancement (by preventing the tree response to be proportional to the rate 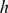) and the screening resonance phenomenon (by blocking forward-propagating dendritic spikes with backward-propagating ones).

With rare exceptions [19],[20], the fact that nonlinear summation often implies spike annihilation has been somewhat underrated in the literature. Recent simulations with biophysical compartments show the propagation and collision of dendritic spikes [19],[20]. To fully evaluate our ideas, one should examine better this phenomenon in *in vitro* dendrites. The computational results suggest the following simple experimental tests:

1. After the simultaneous creation, by two electrodes, of counter propagating spikes on a long apical dendrite, no spike should be detected in either electrode due to spike annihilation in the space between them.
2. Blocking of active channels should reduce the dynamic range of cells with large dendritic arbors, but the effect would be less important in the case of cells with small dendrites (see Fig. 3).
3. After the simultaneous creation of spikes in two dendritic sites on the same subtree, the neuron output should be almost the same as that obtained with injection at a single point (due to spike collision at some branchlet of the subtree). The final output is not the (linear) sum of EPSPs but rather the tree functions as an OR gate if the injected currents are simultaneous and located at a similar level 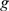.

One consequence of spike annihilation is that under moderate stimulation backspikes will fail to reach more distal branches, owing to collisions with forward-propagating dendritic spikes and/or refractory branches [19],[47]. Indeed, we have observed this phenomenon in our model.

This is compatible with recent observations that backspikes are strongly attenuated in the presence of synaptic input in medial superior olive principal neurons [48]. So, the use of somatic backspikes as a backpropagating signal under massive synaptic input seems to be problematic. Somatic backspikes show up naturally in excitable trees but plays no functional role here.

We conjecture that somatic backspikes may be epiphenomena or perhaps, if they have a functional role in learning processes, it is a recent evolutionary exaptation from previous robust functions like signal amplification by dendritic spikes. This can be tested: our model predicts that active dendrites will be found even in neurons without any plasticity or learning phenomena.

### Relation to psychophysics

Our modeling approach also suggests a microscopic (neural) basis for Stevens law of psychophysics [49],[50], which states that the perception 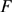 of stimulus intensity 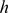 grows as a power law 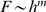. In a previous work with disordered networks [10], by assuming a linear relationship between psychophysical perception and the network activity, we have found a Stevens-like exponent for the input-output function of excitable media with value 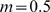. For planar networks we found 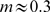 [26]. Here we found for the dendritic tree architecture that the Stevens exponent is very small (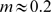 or even 0.1 for large trees with 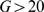), which means that the response function could be confounded with a logarithmic (Weber-Fechner) law [49]. Of course, the macroscopic psychophysical law would be a convolution of all these non-linear transfer functions between the sensory periphery and the final processing (psychological) stage.

What our model shows is that any excitable medium naturally presents a nonlinear input-output response with exponent 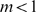, that is, large dynamic range, and that perceptual “psychophysical laws” could be a very early phenomenon in evolution. A simple precondition is that the sensory network should have an excitable spatially extended dynamics, like the one already found in bacterial chemotaxis channel networks, for example [51],[52].

### Model robustness

In our model, variable branchlet diameter and size is described by a spatial dependence and disorder in *p_λ_*. We do not expect the results concerning the dynamic range to change qualitatively with this type of generalization. The same model robustness appears for changes in the refractory time and the use of continuous dynamical variables (maps or differential equations). This latter property has already been demonstrated in multilevel modeling studies which used cellular automata and nonlinear differential equations to describe the neuronal excitable elements [22],[27],[28]. We now explicitly show the results for three variants of the model in order to address the robustness of the dynamic range enhancement.

#### Model I: Propagation asymmetry

First, we consider the possibility that backward transmission of excitation is less likely to occur than in the forward direction (as suggested by impedance matching arguments). For that purpose, we keep 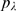 for denoting the probability of transmission in the forward direction and let 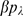 be the probability of transmission in the backward direction, with 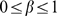.

For 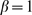, a given active branchlet at level 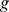 excites a quiescent daughter at 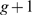 and its quiescent mother at 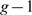 with the same probability (this corresponds to the results presented so far). At the other extreme, for 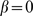, backpropagating dendritic spikes do not occur at all.

In Fig. 5A we show the 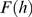 curves for varying *β*, which present pronounced double-sigmoid behavior, suggesting that backspikes regularize the first sigmoidal saturation. In Fig. 5B we see that the dynamic range 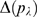 curves are almost the same for different values of 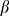 (note that the differences among the 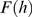 curves occur for intermediate values of 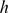, so the 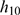 and 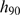 points remain essentially the same). Therefore, a possible functional role for backspikes could be to regularize the response curve, since the first saturation of a double-sigmoid corresponds to poor coding. Of course, this presumed functional role is only speculative, but is a new suggestion provided by our model.

Another result of this variant of the model confirms that backspikes are indeed responsible for the crossing of the response curves 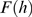 for different values of 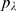 (see Fig. 2B). As depicted in Fig. 4C, the screening resonance phenomenon only occurs for 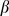 sufficiently close to one.

We also examined the effect of varying the coupling between branchlets (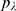, Fig. 5C) and their refractory period (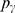, Fig. 5D) in the asymmetric propagation model with 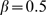. We see some new phenomena like a non-monotonic dependence of 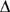 on the coupling 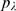 (Fig. 5D), which only occurs for 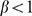.

#### Model II: Non-homogeneous branchlet activation

Since the rate 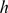 reflects the imbalance between synaptic excitation and inhibition at the branchlets, and since different branchlets may receive a different number of synapses, a step torward more realistic modeling would involve a branchlet-dependent 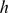. We investigate the effects of a kind of non-homogeneity present in several neurons, where synaptic density or excitability tends to be larger in more distal branchlets.

A simple model that incorporates this spatial dependence is 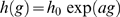. For 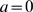 we recover the homogeneous model, while for 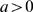 the rate of dendritic spike creation increases with the distance from the soma (note in particular that for 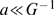 this increase is approximately linear). The use of an exponential model is motivated by the fact that it is an extreme one: any polynomial dependence model lies between the uniform and the exponential case. If the exponential model does not produce qualitative changes, then polynomial models hardly will do.

**Figure 6:**
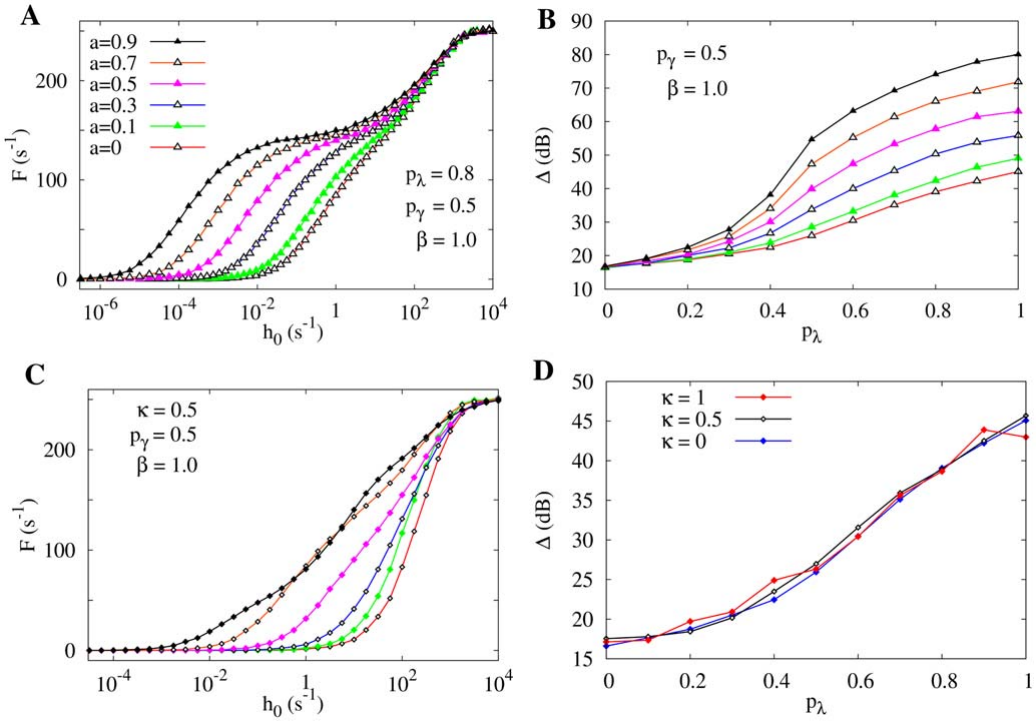
Effect of heterogeneous tree activation. (A) Response functions 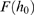 for the exponential activation distribution 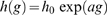 where 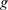 refers to the branchlet generation and 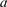 controls the exponential shape. More distal branchlets (larger 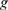) are more activated than the apical site. For large values of parameter 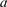 the sensitivity of the response function is greatly increased while the saturation remains almost the same. All curves have 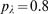, 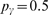 and 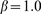. (B) Dynamic range for the previous case with 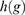 activation, with an amazing enlargement of the dynamic range for 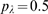 and 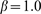. All cases refer to tree sizes of 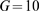. (C) Response functions 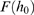 for the disordered branchlet activation model with coefficient of variation 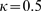, recovery probability 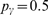, symmetric propagation (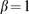) and 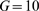. From bottom to top, 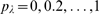. (D) The dynamic range remains the same for this disordered scenario in the tree (same parameters of panel (C)). Note that a coefficient of variation 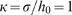 corresponds already to a highly heterogeneous case.

In Fig. 6A we show the response curves 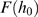 for several values of the parameter 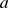. In Fig. 6B we show that the enhancement of dynamic range obtained from the response curves 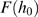 (for different values of 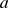) is robust. Surprisingly, the signal amplification and dynamic range is indeed much more efficient than the homogeneous case 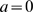, attaining 80 dB (notwithstanding the poor coding for intermediate values of 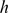, where the size of the plateau increases with 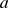 and 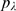). This result suggests that this case of peripheral branchlets being more excitable could optimize the signal processing, specially for neurons with poor propagation of dendritic spikes (small 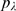, see Fig. 6B).

#### Model III: Disordered branchlet activation rate

In the previous variant, all branchlets in the same generation 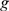 have the same activation rate 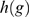. Now, we study a disordered 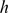 model, where each branchlet 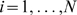 is initially assigned a rate 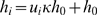. The parameter 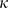 is fixed for each curve and 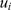 is drawn from a Gaussian distribution with zero mean and unit variance, and is kept constant throughout each run. Note that 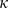 corresponds to the coefficient of variation 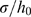 of the distribution 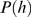, where 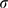 is the standard deviation. Since the Poisson excitation rate 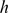 must be positive, we set 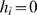 if we some branchlet gets a 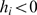 from the Gaussian distribution.

The response curves 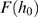 for different values of 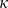 are shown in Fig. 6C. The enhancement of dynamic range is essentially unchanged even under strong variability (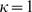) of branchlet activation rate (Fig. 6D).

### Conclusions and perspectives

Several detailed biophysical models of dendritic trees have already been presented in the literature, but we are not aware of studies confirming the enlargement of the dynamic range by active dendrites in such arbors. To see this effect, it is necessary that such models incorporate inputs distributed along the full dendritic tree, and that the branchlet activation rate be varied by orders of magnitude.

We believe that biophysical multi-compartmental models (with a large number of branchlets) seeking to probe the robustness of our results would be most welcome. In particular, they would be able to address the effect of post-synaptic potentials (PSPs, both excitatory and inhibitory) which manage to generate somatic – but not dendritic – spikes despite the presence of active channels in the dendrites (a phenomenon which might be artificially adapted to our model, but for which biophysical models are better equipped). Also, the modulatory effect of such subthreshold PSPs and other passive phenomena are better studied in biophysical simulations.

Other future tasks will be the study of the dendritic response due to non-Poisson input distributions (say, 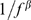 noise), correlated input on the arbor, time-dependent inputs, asymmetric dendritic trees etc. We believe that new signal processing features may appear, but the dynamic range enlargement and sensitivity enhancement by active dynamics will continue to be present.

Why do neurons have active channels in extensive dendritic trees? Our proposal is that active large dendrites are able to detect and amplify very weak signals and, at the same time, saturate slowly for stronger tree activity. This universal “dynamic range” problem, related to the trade-off between sensitivity and saturation of signal processing, is important both for individual neurons, large neural networks, whole sensory organs and organisms. We conjecture that the large dynamic ranges found in neurons with active dendritic arbors could even help to explain macroscopic psychophysical laws, providing a neural account for the century old findings of Fechner, Weber and Stevens [10],[49],[53].
